# Role of epidural ketamine for postoperative analgesia after upper abdominal surgery

**DOI:** 10.4103/0019-5049.79894

**Published:** 2011

**Authors:** Mamta Sethi, Nitin Sethi, Pradeep Jain, Jayashree Sood

**Affiliations:** Department of Anaesthesia, Max Superspeciality Hospital, Saket, New Delhi, India; 1 Department of Anaesthesiology, Pain and Perioperative Medicine, Sir Ganga Ram Hospital, New Delhi, India

**Keywords:** Epidural analgesia, ketamine, morphine, postoperative pain

## Abstract

Ketamine, a *N*-methyl-D-aspartate receptor antagonist inhibits central sensitization due to peripheral nociception thus potentiating the analgesic effect of morphine. The purpose of our study was to evaluate the effect of adding small-dose ketamine in a multimodal regimen of postoperative patient-controlled epidural analgesia (PCEA). One hundred patients of American Society of Anesthesiologists physical status I-II, undergoing major upper abdominal surgery were randomly allocated to two groups. Group I received PCEA device containing bupivacaine hydrochloride 0.0625% and morphine sulphate (preservative free) 0.05mg/ml. Group II received PCEA device containing bupivacaine hydrochloride 0.0625%, morphine sulphate (preservative free) 0.05 mg/ml and ketamine hydrochloride (preservative free) 0.2 mg/ml. The mean morphine consumption in group I after 1^st^and 2^nd^postoperative day was 8.38±2.85 and 7.64±1.95 mg, respectively, compared to 6.81±1.35 and 6.25±1.22 mg (*P*<0.05) in group II. Although group II consumed significantly less morphine, pain relief at rest and at movement after 6, 12, 24 and 48 hours, postoperatively was significantly better in group II (*P*<0.05) than in group I. These findings suggest that adding small-dose ketamine to a multimodal PCEA regimen provides better postoperative analgesia and reduces morphine consumption.

## INTRODUCTION

The use of epidural analgesia for the management of postoperative pain has evolved as a critical component of multimodal approach to achieve the goal of adequate analgesia with improved outcome. Epidural analgesia offers superior postoperative pain relief compared with systemic opioids. In addition to improved pain control, epidural analgesia can improve patient outcome by attenuating detrimental perioperative physiology.[1,2]

A combination of bupivacaine and morphine provides more effective analgesia than morphine used alone, with less opioid-related side-effects.[3,4]

There is evidence that administration of an opioid after tissue injury activates *N*-methyl-D-aspartate (NMDA) receptors, which are ligand-gated ion channels in the spinal dorsal horn, resulting in central sensitization and severe pain.[5] Ketamine, a non-competitive NMDA receptor antagonist, inhibits central sensitization, thus potentiating the analgesic effect of epidural morphine.[6]

The purpose of this study was to examine the effect of adding ketamine in a multimodal patient controlled epidural analgesia (PCEA) regimen for postoperative pain relief in patients undergoing upper abdominal surgery.

## METHODS

After obtaining written informed consent and hospital ethics committee approval, a prospective double blind randomized controlled study was conducted on 100 patients of either sex, aged 18– 65 yrs, belonging to American Society of Anesthesiologists (ASA) physical status classification I-II undergoing major upper abdominal surgery.

The patients were randomly allocated using computer generated numbers into two groups of 50 each.

Group I- Bupivacaine with morphine

Group II- Bupivacaine with morphine plus ketamine

The exclusion criteria were -

1. Contraindication to regional blockade
2. History of opioid addiction
3. History of psychological disorder
4. ASA physical status classification > II

In the preoperative period all patients were instructed about the use of PCEA device and 10-point visual analogue scale (VAS; 0=no pain, 10=worst ever pain).

Prior to induction of anaesthesia an epidural catheter was placed in the T8–T10 intervertebral space in lateral position by using 10-cm long, 18-gauge Tuohy`s needle with loss of resistance technique. The epidural catheter position was rechecked with a test dose comprising 3 ml of 2% lignocaine with adrenaline (5 μg/ml).

In all patients standard general anaesthesia with controlled ventilation was used. Anaesthesia was induced with thiopentone sodium 5 mg/kg and fentanyl citrate 2 μg/kg and trachea intubated with atracurium besylate 0.5mg/kg.

Anaesthesia was maintained with atracurium besylate 0.5 mg/kg/hr and isoflurane 1-2% in air- oxygen mixture to maintain muscle relaxation and depth of anaesthesia, respectively. Intraoperative analgesia was achieved with intermittent boluses of fentanyl citrate 1 μg/kg.

After the end of procedure and reversal of residual neuromuscular blockade with neostigmine methylsulphate 50 μg/kg and glycopyrrolate 10 μg/kg, thepatient was shifted to the post-anaesthesia care unit (PACU). In PACU, the patient was assessed for pain intensity using the 10 point VAS. If VAS ≥3, an initial dose of 0.125% bupivacaine 10 ml was administered via epidural catheter. A PCEA pump was then attached.

Group I:- Bupivacaine with Morphine

This group received PCEA regimen containing bupivacaine hydrochloride 0.0625% and morphine sulphate (preservative free) 0.05 mg/ml.

PCEA pump was programmed to administer basal flow 4 ml/hr, patient-controlled analgesia (PCA) 6 ml/dose with lockout interval of 30 minutes.

Group II:- Bupivacaine with Morphine plus Ketamine

This group received PCEA regimen containing bupivacaine hydrochloride 0.0625%, morphine sulphate (preservative free) 0.05 mg/ml and ketamine hydrochloride (preservative free) 0.2 mg/ml.

PCEA pump was programmed to administer basal flow 4 ml/hr, PCA 6 ml/dose with lockout interval of 30 minutes.

In both groups the following parameters were recorded:

1. Pain at rest and movement, analysed by the 10 point VAS
2. Total amount of morphine consumed
3. Rescue analgesic requirement
4. Sedation was recorded according to a 4-point scale
  0= awake and alert
  1= mildly sedated
  2= moderately sedated, aroused by shaking
  3= deeply sedated, difficult to arouse even by shaking
5. Morphine-induced side-effects - nausea, vomiting and pruritus
6. Ketamine-induced psychomimetic effects - hallucinations and delirium

The patients were assessed at varying time intervals. At 0 hour, pain at rest and at movement was assessed. All other parameters except morphine consumption was assessed at 6, 12, 24 and 48 hours of attachment of PCEA pump. Morphine consumption was assessed at the end of 24 hrs (1^st^ postoperative day) and 48 hrs (2^nd^ postoperative day) of attachment of PCEA pump.

Rescue analgesia was given with injection diclofenac sodium 75 mg via slow intravenous infusion. Nausea and vomiting was treated with injection metoclopramide 10 mg intravenously. Pruritus was treated with injection pheniramine maleate 10 mg intravenously.

Postoperatively if systolic blood pressure < 90 mmHg or if there was any sign of respiratory depression (respiratory rate <10/min) the PCEA pump was discontinued and the patient excluded from the study.

### Statistical analysis

Statistical analysis was done using the SPSS version 11.5. Assuming type I error of 0.05 and a type II error of 0.1 to detect 30% difference in morphine consumption the estimated number of patient in each group was 30. Categorical data was analysed using the χ^2^ test. Parametric data comprising of age, weight and morphine consumption was analyzed using the unpaired t- test. VAS was analyzed using the Mann-Whitney test. A *P* value < 0.05 was considered to be significant.

## RESULTS

The demographic profile in both the groups was comparable [Table 1]. The mean morphine consumption in group I after 1^st^ and 2^nd^ postoperative day was 8.38±2.85 and 7.64±1.95 mg, respectively, compared to 6.81±1.35 and 6.25±1.22 mg in group II [*P*<0.05, Table 2]. The postoperative pain scores at rest and movement were significantly lower (*P*<0.05) in group II as compared to group I at 6, 12, 24 and 48 hours [Tables 3 and 4]. Fourteen (28%) patients in group I compared to 4 (8%) patients in group II had rescue analgesic requirement (*P*=0.009). The incidence of nausea/vomiting and pruritus was more in group I as compared to group II [*P*<0.05, Table 5]. All the patients in both the groups were awake and alert and there was no incidence of psychosis.

**Table 1:** Demographic data

Demographic variables	Group I (n=50)	Group II (n=50)
Age (years)	48.9+13.64	51.44+14.38
Sex (male:female)	34:16	30:20
Weight (kg)	69.34+5.18	70.68+5.98
Height (cms)	161.5+7.17	162.6+6.72

Values expressed as mean±S.D.

**Table 2:** Morphine consumption

	Group I	Group II	*P* value
Morphine consumption (mg) (1^st^ postoperative day)	8.38±2.85	6.81±1.35	0.001
Morphine consumption (mg) (2^nd^ postoperative day)	7.64±1.95	6.25±1.22	0.001

Values expressed as mean± S.D., *P*<0.05 significant.

**Table 3:** Postoperative pain during rest

Visual analogue score during rest (VAS – R)	Group I (n=50)	Group II (n=50)	*P* value
VAS at 0 hrs (VAS – R0)	4.98±1.09	4.56±0.97	0.056
VAS at 6 hrs (VAS – R6)	4.8±1.03	3.8±1.35	0.001
VAS at 12 hrs (VAS - R12)	4.6±0.85	3.64±1.04	0.001
VAS at 24 hrs (VAS - R24)	4.12±0.32	2.3±0.67	0.001
VAS at 48 hrs (VAS - R48)	3.12±0.32	1.46±0.76	0.001

VAS - R0, R6, R12, R24 and R48: Visual analogue score during rest at 0, 6, 12, 24 and 48 hrs, respectively, Values expressed as mean±S.D., *P*<0.05 significant.

**Table 4:** Postoperative pain during movement

Visual analogue score during movement (VAS - M)	Group I (n=50)	Group II (n=50)	*P* value
VAS at 0 hrs (VAS - M0)	5.9±1.03	5.6±0.92	0.130
VAS at 6 hrs (VAS - M6)	5.84±1.01	5.1±0.30	0.001
VAS at 12 hrs (VAS - M12)	5.74±0.96	4.6±0.92	0.001
VAS at 24 hrs (VAS - M24)	5.14±0.35	3.9±1.14	0.001
VAS at 48 hrs (VAS - M48)	3.92±0.87	2.9±1.02	0.001

VAS – M0, M6, M12, MR24 and M48: Visual analogue score during movement at 0, 6, 12, 24 and 48 hrs, respectively, Values expressed as mean± S.D, *P*<0.05 significant.

**Table 5:** Side-effects

Side-effects	Group I (n=50)	Group II (n=50)	*P* value
Sedation	0	0	(a)
Nausea/vomiting	16 (32%)	4 (8%)	0.003
Pruritus	12 (24%)	3 (6%)	0.012
Hallucinations/delirium	0	0	(a)

(a) – no statistics computed, *P*<0.05 significant

## DISCUSSION

This study compared the effectiveness of PCEA in postoperative pain control between patient groups receiving epidural morphine plus local anaesthesia alone or combined with low-dose ketamine. The various drugs used for providing analgesia via the epidural route act at different receptors in the spinal cord which decreases postoperative pain and also the incidence of side-effects.

Morphine is commonly used for providing postoperative epidural analgesia. Morphine is hydrophilic in nature because of which it has a prolonged duration of action and a widespread segmental effect. Epidural analgesia is most commonly provided using a combination of local anaesthetic and an opioid. Compared with opioids or local anaesthetics alone, a local anaesthetic–opioid combination provides superior postoperative analgesia with lower local anaesthetic doses and less opioid-related side-effects.[3,4] It is not clear whether the analgesic effects of the combination of local anaesthetics and opioids are additive or synergistic[4,7] but experimental studies imply a synergistic effect.[8]

Opioids exposure especially in large doses seems to increase release of glutamate from presynaptic terminals within the spinal cord and enhances NMDA receptor-mediated neuronal responses in dorsal horn neurons and in other areas of the central nervous system. Activation of the NMDA receptors produces hyperexcitability of the dorsal horn neurons inducing central sensitization, windup phenomenon and severe pain.[5,9,10] Thus, stimulation of NMDA receptors by morphine may reduce and shorten its antinociceptive effect.

NMDA receptor antagonists inhibit hyperalgesia caused by inflammation, tissue and nerve injury.[11] Clinical studies have shown that epidural administration of ketamine along with morphine reduces postoperative morphine consumption and provides effective analgesia.[12,13]

There have been concerns regarding the neurotoxicity of neuraxial ketamine. Spinal myelopathy has been reported with intrathecal injection of large doses of ketamine.[14] However, single and repeated administration of diluted epidural ketamine has been found to be devoid of neurotoxic effects.[12,15] Preservative-free ketamine in a concenteration of 0.2 mg/ml was used in our study because of our concerns regarding neurotoxicity.

A study[16] done to evaluate the effect of addition of ketamine in a multimodal patient-controlled epidural regimen containing ketamine, bupivacaine and epinephrine in patients undergoing thoracic and upper abdominal surgery concluded that adding small-dose ketamine provides better postoperative analgesia than a PCEA regimen without ketamine especially during cough or movement, thus reducing analgesic consumption. In another study[17] PCEA was administered for lower abdominal surgeries with morphine with or without ketamine and a mean morphine consumption of 8.6 mg was reported in the morphine group in comparison to 6.2 mg in patients receiving ketamine.

The average morphine consumption in group I on postoperative days 1 and 2 was 8.38 and 7.64 mg, respectively, which was higher compared to patients in group II, where the consumption was 6.81 and 6.25 mg, respectively. The additional analgesic requirement was also lower in group II (8%) as compared to group I (28%).

The mean VAS score at rest and at movement at 0 hours was similar in both the groups. VAS score at rest and at movement at 6, 12, 24 and 48 hours was more in group I as compared to group II.

Patients in group I had a higher incidence of nausea/vomiting (32%) and pruritus (24%) compared to group II (8 and 6%, respectively). The higher incidence of nausea/vomiting and pruritus in group I might have been due to the higher total dose of morphine consumption in these patients.

No serious adverse effect, such as respiratory depression or haemodynamic instability were noted in either of the groups. Psychomimetic effects, one of the most troubling side-effects of ketamine, were not observed in any of our patients.

## CONCLUSIONS

A combination of low-dose epidural ketamine and morphine results in effective postoperative analgesia and also reduces morphine requirement, thereby decreasing morphine-related side-effects.
